# Succinate dehydrogenase B-deficient cancer cells are highly sensitive to bromodomain and extra-terminal inhibitors

**DOI:** 10.18632/oncotarget.15959

**Published:** 2017-03-07

**Authors:** Satoshi Kitazawa, Shunsuke Ebara, Ayumi Ando, Yuji Baba, Yoshinori Satomi, Tomoyoshi Soga, Takahito Hara

**Affiliations:** ^1^ Oncology Drug Discovery Unit, Pharmaceutical Research Division, Takeda Pharmaceutical Company Limited, Fujisawa, Kanagawa, 251-8555, Japan; ^2^ Integrated Technology Research Laboratories, Pharmaceutical Research Division, Takeda Pharmaceutical Company Limited, Fujisawa, Kanagawa, 251-8555, Japan; ^3^ Institute for Advanced Biosciences, Keio University, Tsuruoka, Yamagata, 997-0052, Japan

**Keywords:** SDHB, metabolism, c-Myc, BET, TCA cycle

## Abstract

Mutations in succinate dehydrogenase B (SDHB) gene are frequently observed in several tumors and associated with poor prognosis in these tumors. Therefore, drugs effective for SDHB-deficient tumors could fulfill an unmet medical need. In addition, such drugs would have an advantage in that selection of patients with SDHB-mutant cancer could increase the probability of success in clinical trials. Currently, however, the characteristics of SDHB-deficient cancers are not completely understood. Here, we established SDHB knockout cancer cell lines from human colon cancer HCT116 cells using the clustered regularly interspaced short palindromic repeat (CRISPR)/Cas9 knockout system, and clarified its metabolic characteristics.

In the SDHB knockout cells, succinate was accumulated and fumarate was decreased. The oxygen consumption rate was decreased while the extracellular acidification rate was increased in the SDHB knockout cells. Accordingly, an enhanced glycolysis pathway in the SDHB knockout cells was demonstrated by metabolomics analysis. Tracer experiments showed bidirectional metabolic flow in the tricarboxylic acid (TCA) cycle, possibly to maintain the necessary amounts of metabolites in the SDHB knockout cells. The proliferation of SDHB knockout cells was suppressed by a glycolysis inhibitor but not by a mitochondrial inhibitor. Additionally, partial dependence on glutaminolysis was observed in the SDHB knockout cells. Compound screening revealed that a bromodomain and extra-terminal (BET) inhibitor, which downregulated c-Myc, suppressed the growth of the SDHB knockout cells more potently than that of control cells. These findings provide an understanding of the metabolic characteristics of SDHB-deficient cancer and its vulnerabilities, which may lead to new therapeutic options.

## INTRODUCTION

Succinate dehydrogenase (SDH) is a mitochondrial enzyme complex composed of four subunits: SDHA, SDHB, SDHC, and SDHD. SDH is an important molecule that functions both in the mitochondrial tricarboxylic acid (TCA) cycle and in the electron transport chain enzyme complex, mitochondrial complex II. Inactivating missense mutations of the genes that encode the SDH subunits are found to cause familial pheochromocytoma and familial paraganglioma [1–6]. These SDH gene alterations, which lead to the loss of enzyme activity and expression, are also observed frequently in several tumors such as renal cell carcinoma, gastrointestinal stromal tumors, colorectal cancer, gastric cancer, and ovarian cancer [7–16]. Thus, SDH subunit genes are considered to be tumor suppressor genes. SDHB mutations, the most frequently detected among the four subunits of SDH [10, 17], are associated with malignancy [5] and poor prognosis [6]. However, effective therapies for SDH-deficient cancer have not yet been established. Therefore, drugs that suppress progression of cancer with an SDHB deficiency could fulfill an unmet medical need. In addition, such drugs would have an advantage in that selection of patients with SDHB mutant cancer could increase the probability of success in the clinical development of the drugs. To develop drugs that are effective against such tumors requires deep understanding of the characteristics of SDHB-deficient cancer. However, it has not yet been fully understood how SDHB-null cancer changes intracellular metabolism and adapts for survival.

It has been reported that activated pyruvate carboxylase (PC) supplements glucose-derived carbon sources in aspartate synthesis in SDHB knockout cells, and SDHB knockout cell growth is dependent on PC [18, 19]. However, these previous studies used mouse models of normal cells. Therefore, it is not fully understood how human cancer cells, which completely ablate SDHB expression, change intracellular metabolism and adapt for survival.

In this study, we established SDHB knockout cancer cell lines from the human colon cancer HCT116 cell line, and characterized them to identify vulnerabilities that could be targeted therapeutically in SDHB-deficient cancer.

## RESULTS

### SDHB-deficient cells exhibit reduced aerobic metabolism and enhanced glycolysis

**Figure 1:**
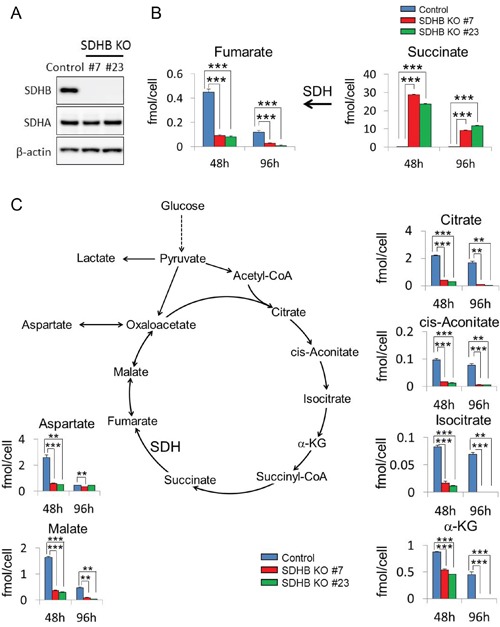
Inhibition of aerobic metabolism in SDHB knockout cancer cells **(A)** Cells were lysed 64 h after cell seeding, and expression levels of SDHB, SDHA, and β-actin were determined by western blotting. **(B, C)** Metabolite concentrations of fumarate, succinate, aspartate, malate, citrate, cis-aconitate, isocitrate, and α-ketoglutarate (α-KG) 48 and 96 h after cell seeding in HCT116 SDHB knockout cells (#7 and #23) compared with control cells, assessed using CE-MS (n = 3). Data are given as means ± standard deviation (SD). *P < 0.05, **P < 0.01, ***P < 0.001 by the Bonferroni's corrected *t*-test.

As described in the materials and methods section, SDHB knockout cells #7 and #23 were generated which completely lost protein expression of SDHB (Figure 1A). It was also confirmed that expression of another subunit, SDHA, was not affected in either SDHB knockout #7 or SDHB knockout #23 cells (Figure 1A). The growth rate of established SDHB-deficient cells was approximately three times lower than that of control cells (Supplementary Figure 1A). Clear accumulation of succinate, a substrate of SDH, and a significant decrease in levels of fumarate, a product of SDH, were revealed by metabolomics analysis in SDHB knockout cells (Figure 1B). Levels of all other metabolites in the TCA cycle such as malate, citrate, isocitrate, cis-aconitate, and α-KG were also decreased (Figure 1C), indicating inhibition of aerobic metabolism in SDHB knockout cells.

**Figure 2:**
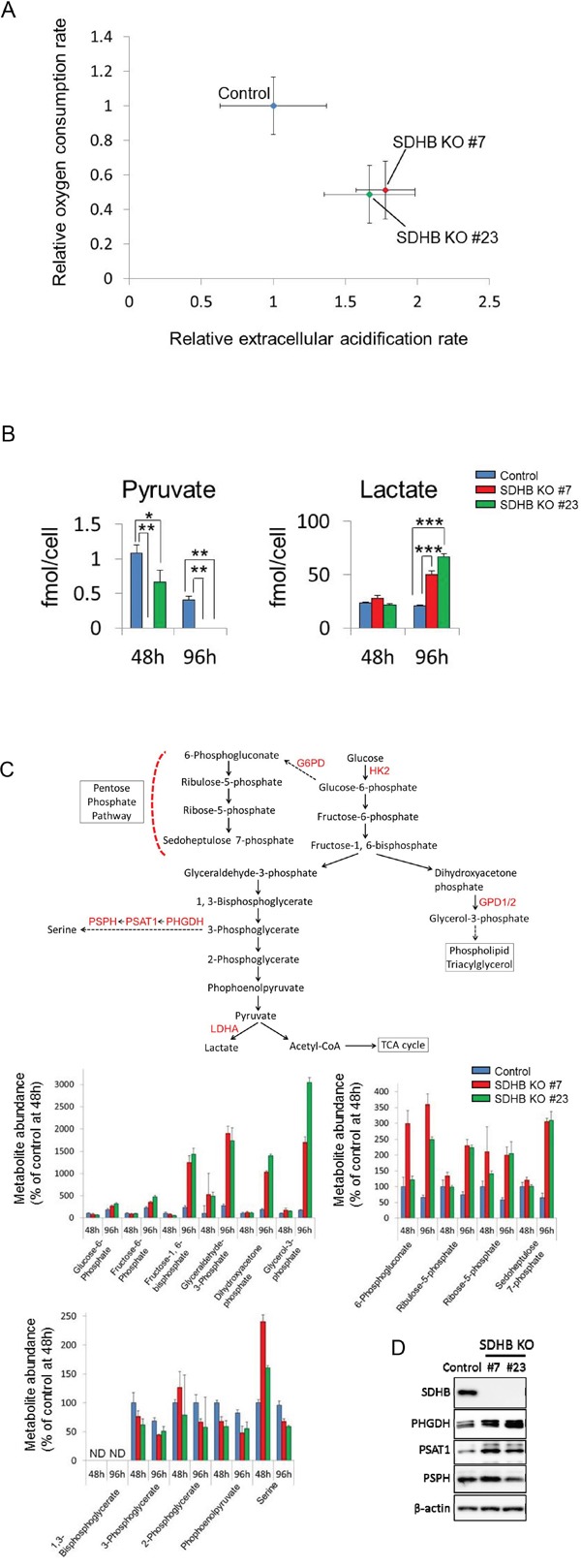
Metabolic switch from aerobic metabolism to glycolysis in SDHB-deficient cells **(A)** Relative values of OCR and ECAR of HCT116 SDHB knockout cells (#7 and #23) were obtained by setting the OCR and ECAR values of the control cells as 1. The OCR and ECAR were analyzed using an XF96 extracellular flux analyzer (n = 12). Data are given as means ± SD. **(B)** Metabolite concentrations of pyruvate and lactate were measured using CE-MS 48 and 96 h after cell seeding in HCT116 SDHB knockout cells (#7 and #23) and control cells (n = 3). Data are given as means ± SD. *P < 0.05, **P < 0.01, ***P < 0.001 by the Bonferroni's corrected *t*-test. **(C)** Metabolites and enzymes in the glycolysis pathway and its derived pathways. Metabolite abundance 48 h after cell seeding was analyzed using CE-MS (n = 3). Ordinate values were obtained by setting the control group value 48 h after cell seeding as 100%. Data are given as means ± SD. ND: not detected. ND: the metabolite concentration was below the detection limit of the analysis. **(D)** Expression levels of SDHB, PHGDH, PSAT1, PSPH, and β-actin were determined by western blotting 64 h after cell seeding.

In SDHB knockouts #7 and #23, the oxygen consumption rate (OCR) was decreased, while the extracellular acidification rate (ECAR) was enhanced compared with control cells (Figure 2A). When SDHB knockouts #7 and #23 were cultured until confluence, the medium turned yellow (Supplementary Figure 1B), indicating a lowered pH, possibly because the accumulated intracellular lactate was excreted into the medium. Consistent with this, remarkable levels of accumulation of intracellular and extracellular lactate, as well as reduced intracellular pyruvate levels, were revealed by metabolomics analysis in SDHB knockout cells (Figure 2B and Supplementary Figure 1C), suggesting that glycolysis may be more strongly activated in SDHB knockout cells compared with control cells. Indeed, greater increases in metabolites were observed upstream of glycolytic metabolism and in the pentose phosphate pathway (PPP) (Figure 2C). Levels of glucose-6-phosphate, fructose-6-phosphate, fructose-1, 6-bisphosphate, glyceraldehyde-3-phosphate, 6-phosphogluconate, ribulose-5-phosphate, ribose-5-phosphate, and sedoheptulose 7-phosphate were clearly increased in SDHB knockout cells (Figure 2C), whereas levels of metabolites downstream from 3-phophoglycerate in glycolysis such as 3-phophoglycerate, 2-phosphoglycerate, and phosphoenolpyruvate were scarcely altered (Figure 2C). In addition, levels of dihydroxyacetone phosphate and glycerol-3-phosphate were substantially increased (Figure 2C), suggesting an enhanced phospholipid synthesis pathway from glycolysis in SDHB knockout cells. Serine levels were also strongly increased at 48 h in SDHB knockout cells (Figure 2C), and expression levels of the serine biosynthesis enzymes phosphoglycerate dehydrogenase (PHGDH) and phosphoserine aminotransferase 1 (PSAT1) were higher in SDHB knockouts #7 and #23 than in control cells (Figure 2D), indicating the perturbed regulation of serine biosynthesis in the SDHB-deficient cells. Taken together, these results suggest that SDHB-deficient cells have reduced mitochondrial aerobic metabolism and therefore their glycolytic metabolism is enhanced, as well as the derivative metabolic pathways, in a compensatory manner.

### Growth of SDHB-deficient cells was dependent on glycolysis

**Figure 3:**
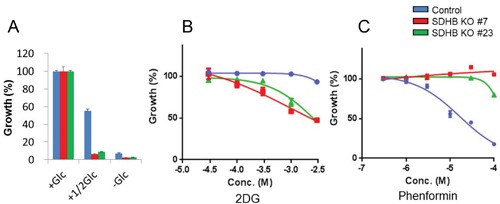
Growth of SDHB knockout cells dependent on glycolysis **(A)** Growth rate of SDHB knockout cells and control cells cultured under conditions of regular glucose concentration (2 g/L), half glucose concentration (1 g/L), and glucose deprivation (0 g/L). **(B, C)** SDHB knockout cells and control cells were exposed to 2DG and phenformin. After 4 days, cell viability was assessed. Ordinate values were obtained by setting the control group value as 100%. Data are given as means ± SD (n = 3).

Next, we examined whether the proliferation of SDHB knockout cells was dependent on glycolytic metabolism or not. SDHB knockout cells exhibited a lower growth rate in low glucose conditions than did control cells (Figure 3A). In addition, SDHB knockout cells were more susceptible to 2-deoxy-glucose (2DG), a glucose analog that inhibits glycolysis by hexokinase 2 inhibition [20, 21], than control cells (Figure 3B). On the other hand, the growth of SDHB knockout cells was not affected by phenformin, a mitochondrial complex I inhibitor (Figure 3C), while phenformin strongly attenuated the growth of control cells (Figure 3C).

Because the PPP, the phospholipid synthesis pathway, and the serine synthesis pathway were altered in the SDHB knockout cells, we tested the dependence of cell growth on these pathways using inhibitors of each pathway. Susceptibility to a PPP inhibitor (a glucose-6-phosphate dehydrogenase inhibitor, dehydroepiandrosterone) or an inhibitor of the phospholipid synthesis pathway (a glycerol-3-phosphate dehydrogenase 1/2 inhibitor, 5-pentadecylresorcinol) did not differ between SDHB knockout cells and control cells (Supplementary Figure 2A, 2B). SDHB knockout cells showed slightly less sensitivity to an inhibitor of the serine synthesis pathway (a PHGDH inhibitor, CBR-5884) (Supplementary Figure 2C) than control cells. In addition, sensitivity to NHI-2, an inhibitor of lactate dehydrogenase A, which converts pyruvate to lactate, did not differ between SDHB knockout cells and control cells (Supplementary Figure 2D). These results indicate that proliferation of SDHB knockout cells depends on the glycolytic pathway, but not the PPP, the phospholipid synthesis pathway, the serine biosynthesis pathway, or the lactate synthesis pathway.

### Bidirectional carbon flow in the TCA cycle to maintain metabolite levels in SDHB knockout cells

**Figure 4:**
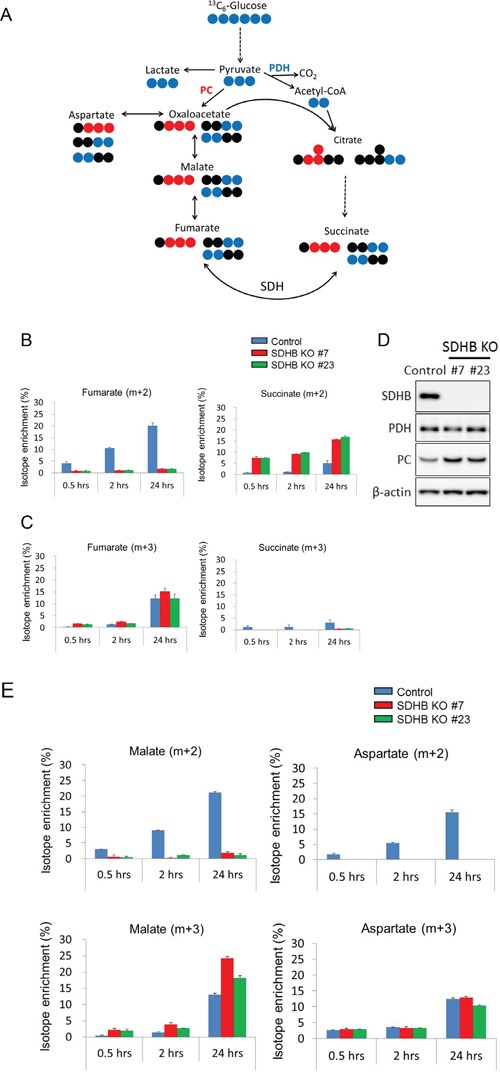
Bidirectional carbon flow in the TCA cycle in SDHB-deficient cells **(A)** The schematic illustration shows ^13^C_6_-glucose entry into the TCA cycle via PC (red) and PDH (blue). The blue and red symbols (●, ●) represent ^13^C atoms and the black symbols (●) represent ^12^C atoms. **(B, C)** ^13^C_6_-glucose incorporated into fumarate and succinate via PC and PDH in SDHB knockout cells and control cells is shown as isotope enrichment of total metabolites. Data are given as means ± SD. **(D)** Cells were lysed 64 h after seeding, and expression levels of SDHB, PDH, PC, and β-actin were determined by western blotting. **(E)** ^13^C_6_-glucose incorporated into malate and aspartate via PC and PDH in SDHB knockout cells and control cells is shown as isotope enrichment of total metabolites. Data are given as means ± SD.

We investigated how carbon sources derived from glucose were utilized in the TCA cycle in SDHB knockout cells using stable isotope-labeled ^13^C_6_-glucose. Glucose-derived pyruvate enters the TCA cycle through two distinct pathways: via pyruvate dehydrogenase (PDH) or PC, as shown in Figure 4A. Enrichment of succinate (m + 2), a metabolite derived from pyruvate via PDH, increased with time in both SDHB knockout cells and control cells (Figure 4B), indicating the presence of carbon flow into the TCA cycle via PDH. As expected from SDH status, fumarate (m + 2), the product of SDH from pyruvate via PDH, was clearly reduced, and succinate (m + 2), the substrate of SDH from pyruvate via PDH, was markedly increased in SDHB-deficient cells compared with control cells (Figure 4B). On the other hand, enrichment of succinate (m + 3), the product of SDH from pyruvate via PC, was detected only slightly in the SDHB knockout cells (Figure 4C), indicating only a little carbon flow into the TCA cycle via PC in a clockwise direction on the diagram in Figure 4A. Interestingly, however, enrichment of fumarate (m + 3) increased with time in both SDHB knockout cells and control cells (Figure 4C), indicating the presence of carbon flow into the TCA cycle via PC in a counterclockwise direction. There was similar or comparatively greater enrichment of malate (m + 3) and aspartate (m + 3) in SDHB knockout cells compared with control cells (Figure 4E). Importantly, the protein expression level of PC was increased in SDHB knockout cells compared with control cells (Figure 4D) while the PDH expression level did not differ between the SDHB knockout cells and control cells (Figure 4D). Taken together, these findings suggest that SDHB knockout cells offset the influence of SDH dysfunction by causing a bidirectional carbon flow in the TCA cycle by enhancing PC function in a counterclockwise direction to maintain the minimum necessary amounts of metabolites.

### Growth of SDHB-deficient cells was dependent on glutaminolysis

**Figure 5:**
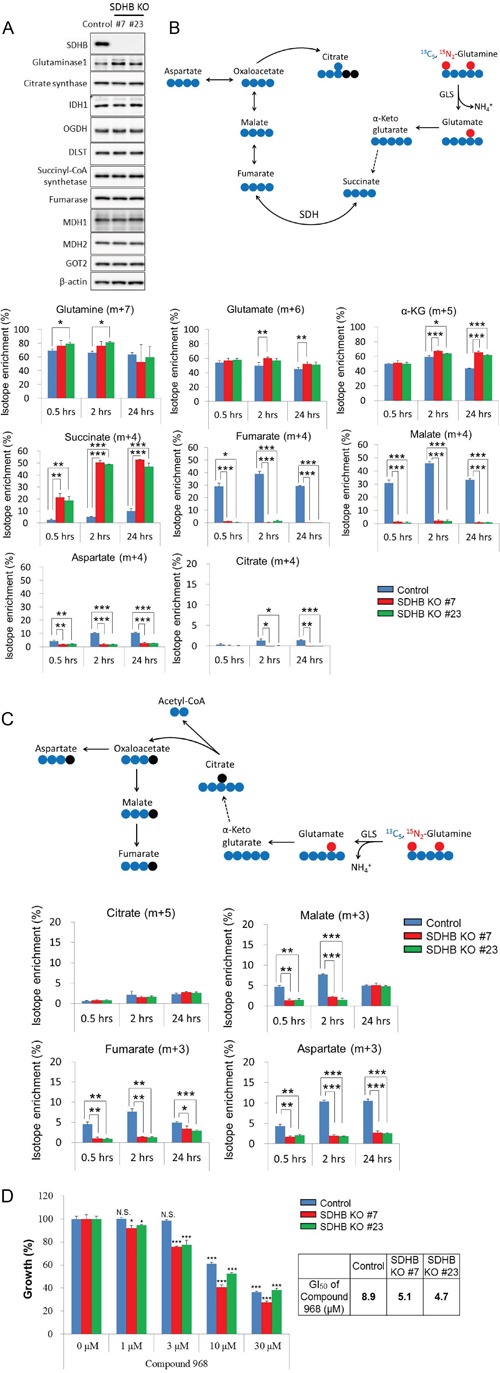
Growth of SDHB knockout cells dependent on glutaminolysis **(A)** Cells were lysed 64 h after seeding, and expression levels of SDHB, glutaminase 1, citrate synthetase, IDH1, OGDH, DLST, succinyl-CoA, fumarase, MDH1, MDH2, GOT2, and β-actin were determined by western blotting. **(B)** The schematic illustration shows ^13^C_5_, ^15^N_2_-glutamine-derived oxidative glutaminolysis. The blue and red symbols (●, ●) represent ^13^C atoms and the black symbols (●) represent ^12^C atoms. Incorporation of ^13^C_5_, ^15^N_2_-glutamine into glutamate, α-ketoglutarate, succinate, fumarate, malate, aspartate, and citrate by oxidative glutaminolysis in SDHB knockout cells and control cells is shown as isotope enrichment of total metabolites. Data are given as means ± SD. *P < 0.05, **P < 0.01, ***P < 0.001 by the Bonferroni's corrected *t*-test. **(C)** The diagram represents ^13^C_5_, ^15^N_2_-glutamine-derived reductive carboxylation. The blue and red symbols (●, ●) represent ^13^C atoms and the black symbols (●) represent ^12^C atoms. Incorporation of ^13^C_5_, ^15^N_2_-glutamine into citrate, malate, fumarate, and aspartate by reductive carboxylation in SDHB knockout cells and control cells is shown as isotope enrichment of total metabolites. Data are given as means ± SD. *P < 0.05, **P < 0.01, ***P < 0.001 by the Bonferroni's corrected *t*-test. **(D)** SDHB knockout cells and control cells were treated with the indicated concentrations of the glutaminase 1 inhibitor Compound 968. After 4 days, cell viability was assessed. Ordinate values were obtained by setting the vehicle control value as 100%. Data are given as means ± SD (n = 3). Growth inhibition of 50% (GI_50_) was calculated using GraphPad Prism version 6 software. N.S., not significant. *P < 0.025, **P < 0.005, ***P < 0.0005 by Williams’ test.

Since increased protein expression of glutaminase 1 among other TCA cycle-related enzymes was found in SDHB knockout cells (Figure 5A), we hypothesized that SDHB knockout cells acquired a higher rate of glutaminolysis. To test this hypothesis, we investigated whether the SDHB-deficient cells used glutamine as an energy source in the TCA cycle by carrying out tracer experiments with a stable isotope-labeled glutamine tracer (^13^C_5_, ^15^N_2_-glutamine). Slightly higher but significant fractional enrichments of glutamine (m + 7), glutamate (m + 6), and α-ketoglutarate (m + 5) derived from ^13^C_5_, ^15^N_2_-glutamine were observed from 0.5 to 24 h after treatment with the labeled glutamine in SDHB knockout cells (Figure 5B). Succinate (m + 4) was remarkably enriched in SDHB-deficient cells (Figure 5B), whereas the SDH product fumarate (m + 4) and its downstream metabolites malate (m + 4), aspartate (m + 4), and citrate (m + 4) were detected only slightly in SDHB knockout cells (Figure 5B). These data indicate that oxidative glutaminolysis was activated in SDHB knockout cells up to succinate production, and its downstream flow was shut down due to the deficiency of SDHB.

Next, we considered the possibility of activation of the reductive carboxylation pathway in SDHB-deficient cells. However, the fractional enrichment of citrate (m + 5) in SDHB knockout cells was slightly increased only in SDHB knockout #7 at 24 h, and isotope enrichment at other time points was not significantly different among control cells and SDHB knockout cells (Figure 5C). Isotope enrichments of the other metabolites such as malate (m + 3), fumarate (m + 3), and aspartate (m + 3) were markedly decreased in SDHB knockout cells (Figure 5C), indicating that the reductive carboxylation pathway was inactivated in SDHB-deficient cells.

We next investigated the dependence of SDHB-deficient cells on glutaminolysis. SDHB knockout cells showed a higher sensitivity to selective glutaminase 1 inhibitors, Compound 968 and CB-839, than the control cells (Figure 5D and Supplementary Figure 3). These results suggest that the growth of SDHB knockout cells is dependent on oxidative glutaminolysis.

### SDHB knockout cells were highly sensitive to BET inhibitors

**Figure 6:**
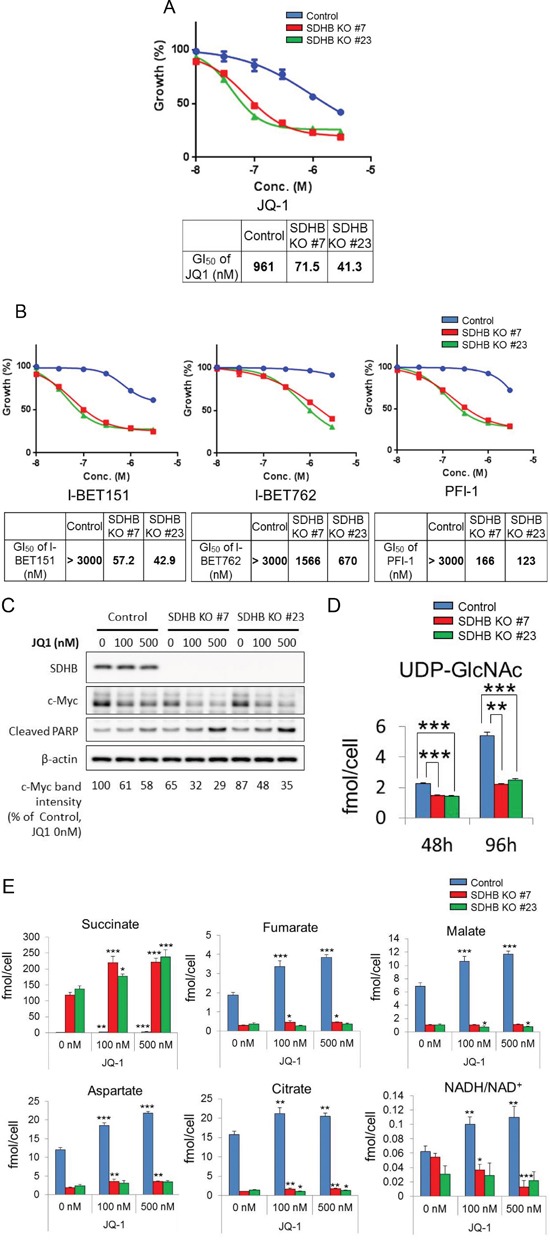
Specific growth inhibition of SDHB-deficient cells by BET inhibition **(A, C)** SDHB knockout cells and control cells were treated with the indicated concentrations of the BET inhibitors JQ-1, I-BET151, I-BET762, and PFI-1. After 4 days, cell viability was assessed. Ordinate values were obtained by setting the vehicle control value as 100%. Data are given as means ± SD (n = 3). Growth inhibition of 50% (GI50) was calculated using GraphPad Prism version 6 software. **(B)** Cells were treated with JQ-1 for 38 h and lysed to detect expression levels of SDHB, c-Myc, cleaved PARP, and β-actin by western blotting. **(D)** Metabolite concentrations of UDP-GlcNAc 48 and 96 h after seeding were assessed using CE-MS (n = 3). Data are given as means ± SD. *P < 0.05, **P < 0.01, ***P < 0.001 by the Bonferroni's corrected t-test. **(E)** SDHB knockout cells and control cells were treated with JQ-1. After 48 h, metabolite concentrations of succinate, fumarate, malate, aspartate, citrate, and NADH/NAD^+^ were assessed using CE-MS (n = 3). Data are presented as means ± SD; N.S., not significant, *P < 0.025, **P < 0.005, ***P < 0.0005 by Williams’ test.

Compound screening revealed that a bromodomain and extra-terminal (BET) inhibitor, JQ1, suppressed the growth of the SDHB knockout cells more potently than that of the control cells (Figure 6A). This seems reasonable, because JQ1 is known as a downregulator of c-Myc [22] and c-Myc is reported to control glycolysis and glutaminolysis [23, 24]. JQ1 suppressed the growth of SDHB knockout cells 13 to 23 times more potently than that of control cells (Figure 6A). It was also confirmed that different chemotypes of BET inhibitors, I-BET151, I-BET762, and PFI-1, also inhibited the growth of SDHB knockout cells more potently than that of control cells (Figure 6B). Cleaved poly-(adenosine diphosphate ribose) polymerase (PARP), a marker of apoptosis induction, was induced more clearly in SDHB knockouts #7 and #23 than in control cells (Figure 6C). We also confirmed that SDHB knockdown by siRNA enhanced the susceptibility to JQ-1 in a renal cell carcinoma cell line Caki-2 (Supplementary Figure 4A, 4B).

Next, we studied the link between c-Myc regulation and growth inhibition by a BET inhibitor in SDHB knockout cells. Protein expression of c-Myc in SDHB knockouts #7 and #23 was downregulated compared with control cells (Figure 6C). In addition, c-Myc expression was more clearly attenuated by JQ1 in SDHB-deficient cells than in control cells (Figure 6C). c-Myc is reportedly stabilized by O-GlcNAcylation at Thr58, for which uridine diphosphate (UDP)-N-acetylglucosamine (UDP-GlcNAc) is utilized [25, 26]. We found that the level of UDP-GlcNAc was reduced in SDHB knockout cells (Figure 6D), which may lead to lower basal c-Myc levels or downregulated c-Myc expression when SDHB knockout cells are treated with a BET inhibitor.

We next examined the metabolite changes induced by JQ-1 treatment in control cells and SDHB knockout cells. There was no significant difference between control cells and SDHB-knockout cells in terms of the glycolysis pathway (data not shown). However, the levels of metabolites in the TCA cycle, such as fumarate, malate, aspartate, and citrate, were increased following JQ-1 treatment more clearly in control cells than in SDHB-knockout cells (Figure 6E). During the TCA cycle, nicotinamide adenine dinucleotide (NADH) was produced using the oxidized form of NADH (NAD^+^) as a substrate. The NADH/NAD^+^ ratio was increased in control cells, but decreased or was comparable in SDHB knockout cells (Figure 6E).

## DISCUSSION

In this study, we showed SDHB-deficient cancer cells exhibit a switch in cellular metabolism, from oxidative phosphorylation to glycolysis, to adapt for survival. In addition to these findings, our tracer experiments with labeled glucose demonstrated that SDHB knockout cells metabolized pyruvate by both PDH and PC to produce succinate and fumarate, respectively, suggesting that SDHB knockout cells offset the influence of SDH dysfunction by causing bidirectional carbon flow in the TCA cycle so as to maintain the necessary amount of metabolites. We also showed increased levels of PC expression in SDHB knockout cells. These results are consistent with previous reports from Cardaci et al. and Lussey-Lepoutre et al. [18, 19]. However, the contribution of glucose-derived carbon sources to aspartate (m + 3) via PC in our SDHB-deficient cancer cells was not as substantial as that reported in their work, which insisted on the importance of PC-dependent aspartate synthesis for SDHB-deficient cancer cell growth.

In our SDHB knockout cells, reductive carboxylation of glutamine was suppressed, but oxidative glutaminolysis up to succinate production was activated regardless of the shutdown of its downstream flow due to the deficiency of SDHB. Consistent with this, the glutaminase 1 expression level was increased in SDHB knockout cells compared with that in control cells. Conversion from glutamine to succinate in glutaminolysis involves several enzymatic reactions to produce adenosine triphosphate (ATP). One molecule of NADH is produced in the reaction by α-ketoglutarate dehydrogenase to generate succinyl-CoA from α-ketoglutarate, and the NADH enters into the mitochondrial electron transport system to synthesize three molecules of ATP. In addition, ATP is also produced during the reaction that produces succinate from succinyl-CoA, which is catalyzed by succinyl-CoA synthetase. ATP production by α-ketoglutarate dehydrogenase and succinyl-CoA synthetase in oxidative glutaminolysis may be necessary for the survival of SDHB-defective cells, because they are inefficient at producing ATP in the TCA cycle due to the loss of SDH activity. Indeed, SDHB knockout cells showed higher sensitivities to the glutaminase 1 inhibitor Compound 968 and CB-839 than did control cells.

Compound screening revealed that a BET inhibitor, JQ1, suppressed the growth of the SDHB knockout cells more potently than that of the control cells. Several chemotypes of BET inhibitors also showed clear specificity for SDHB-deficient cells, which reduced the possibility of a chemical structure-derived unknown off-target effect. We speculate that this specificity of the BET inhibitor is related to c-Myc regulation, because it was reported that a BET inhibitor downregulated c-Myc expression and that c-Myc transcriptionally regulated expression of the genes responsible for glycolysis and glutaminolysis [23, 24].

Increases in levels of metabolites of the TCA cycle, such as fumarate, malate, aspartate, citrate, and NADH/NAD^+^, following JQ-1 treatment were more clearly observed in control cells than in SDHB-knockout cells. These data suggest that activation of TCA cycle may occur as a rescue reaction in control cells following JQ-1 treatment. However, in SDHB-knockout cells, the rescue reaction may be too low to reach adequate levels because of the lower basal levels of the metabolites in SDHB-knockout cells, or the rescue reaction may not occur owing to TCA dysfunction. This may account for the specific inhibition of growth of SDHB-knockout cells by BET inhibitors.

We demonstrated that a BET inhibitor decreased c-Myc expression more clearly in SDHB knockout cells than in control cells. Interestingly, our data also demonstrated lower basal c-Myc protein levels in SDHB knockout cells than in control cells. Lower basal c-Myc levels together with a higher sensitivity of c-Myc downregulation caused by a BET inhibitor may lead to stronger growth inhibition by a BET inhibitor in SDHB knockout cells. The lower basal c-Myc levels may be related to enhanced glycolysis in SDHB knockout cells. It was reported that c-Myc stability was increased by O-GlcNAcylation at Thr58 by O-linked β-N-acetylglucosamine (O-GlcNAc) transferase [25, 26], and that UDP-GlcNAc was synthesized from glucose for this O-GlcNAcylation to stabilize c-Myc [26]. It was also reported that glucose deprivation decreased c-Myc protein stability in certain types of cancer cells [27]. SDHB knockout cells consumed more glucose for activated glycolysis. Therefore, O-GlcNAcylation and stability of c-Myc may be downregulated due to the lack of UDP-GlcNAc in SDHB knockout cells. Indeed, UDP-GlcNAc levels were significantly reduced in SDHB knockout cells. It would be useful to further investigate the precise mechanism of action of BET inhibitors in the growth suppression of SDHB-deficient cancer.

SDHB mutation or deficiency has been detected with high frequency in renal cell carcinoma [10, 11, 17]. In the present study, we confirmed enhanced susceptibility of SDHB-silenced kidney cancer cells to the BET inhibitor JQ-1. Therefore, the specific effect of JQ-1 on SDHB-deficient cells may not be limited to colorectal cancer.

In conclusion, we elucidated the metabolic properties of SDHB-deficient cancer cells and found dependence on glycolysis and glutaminolysis for growth. We identified these metabolic pathways as a vulnerability that can be targeted by BET inhibitors. BET inhibitors may be promising therapeutic agents for SDHB-deficient cancers.

## MATERIALS AND METHODS

### Cell lines

Human colorectal carcinoma cell line HCT-116 and human clear cell renal cell carcinoma line Caki-2 were purchased from the American Type Culture Collection (Manassas, VA, USA). HCT-116 cells were maintained in McCoy's 5A (Gibco) with 10% fetal bovine serum (FBS) (HyClone, SH30071.03), and Caki-2 cells were maintained in RPMI-1640 (Wako, 189-02025) with 10% FBS in an atmosphere of 95% air and 5% CO_2_ at 37°C.

### Generation of SDHB knockout cells

HCT-116 cells were plated at 15000 cells/3 mL/well on 6-well plates (Corning) and cultured overnight. The next day, the cells were transfected with 1 μg of SDHB clustered regularly interspaced short palindromic repeats (CRISPR)/Cas9 knockout plasmid (Santa Cruz) and SDHB homology directed repair (HDR) plasmid (Santa Cruz) or a control CRISPR/Cas9 plasmid (Santa Cruz) using Lipofectamine 2000 (Thermo Fisher Scientific) as described by the manufacturer. After forty-eight hours post transfection, cells were selected with 2 μg/μL of puromycin (Sigma) for 1 week. Cells transfected with the SDHB knockout plasmid were collected as SDHB knockout polyclonal cells. Cells were cloned by limited dilution on 96-well plates (Corning), and cloned cells were collected for analysis of protein expression and genome copy number of SDHB. After transfection of SDHB knockout plasmid, 1 mM sodium pyruvate (Gibco) and 50 μg/ml of uridine (Sigma) were added to the culture medium described above because cells that lack a functional mitochondrial electron transport chain such as ρ0 cells fail to proliferate without supra-physiological levels of uridine and pyruvate in the culture medium [28].

### Confirmation of genomic status of SDHB in SDHB knockout cells

The number of copies of the SDHB exon 2 knockout sites in the genome of two selected single clones of homologous SDHB knockout cells (SDHB knockouts #7 and #23) were examined by quantitative polymerase chain reaction (qPCR). Compared with HCT116 cells transfected with the control vector (control cells), SDHB knockouts #7 and #23 contained half the number of copies of SDHB (Supplementary Figure 5A). A slight decrease in SDHB was observed in the original polyclonal SDHB knockout cells, which were a mixed population of SDHB knockout plasmid-transfected and untransfected cells (Supplementary Figure 5A). The number of copies of SDHB exon 3, which was not a knockout site of this plasmid, was unaltered among the respective cells, and the puromycin resistance gene inserted by the HDR plasmid was detected only in SDHB knockouts #7 and #23 (Supplementary Figure 5A). The genome of the SDHB exon 2 knockout site was amplified by PCR and the amount of amplified product was confirmed by agarose gel electrophoresis. This showed that the genome of the SDHB exon 2 knockout site was reduced by about half in SDHB knockouts #7 and #23 compared with control cells (Supplementary Figure 5B). We examined the genome sequence of SDHB exon 2 in the HCT116 parental cells, control cells, SDHB knockout #7, and SDHB knockout #23 by sequencing. This revealed that an adenine was inserted between the 62nd and 63rd base in SDHB knockouts #7 and SDHB knockout #23. Consequently, a termination codon (TAA) was produced in SDHB exon 2 in the SDHB knockouts (Supplementary Figure 5C). The figure shows that the HDR plasmid was inserted at the knockout site of SDHB exon 2 in one allele and the termination codon was accidentally formed in the other allele (Supplementary Figure 5D).

### Extraction of genomic DNA and genome copy number analysis of SDHB

Genomic DNA was extracted from cultured cells using a QIAmp DNA Mini Kit (Qiagen) following the manufacturer's instructions, and quantified using an ND-1000 spectrophotometer (NanoDrop Technologies). Fifty nanograms of genomic DNA were mixed with TaqMan Universal PCR Master Mix (Applied Biosystems) and 0.9 pmol each of a primer and reporter probe designed with the Custom TaqMan Assay Design Tool (Applied Biosystems, https://www5.appliedbiosystems.com/tools/cadt/). For the SDHB exon 2 knockout site the primer sequences were forward 5′-CCCCGTATCAAGAAATTTGCCATCT-3′ and reverse 5′-GTCTGCATATGAGGTTTGTCTCCA-3′, and the reporter probe sequence was 5′-ATCGAT GGGACCCAGACAA-3′. For SDHB exon 3, the primer sequences were forward 5′-CCCCATGGTA TTGGATGCTTTAATC-3′ and reverse 5′-CTCTGCATG ATCTTCGGAAGGT-3′, and the reporter probe sequence was 5′-AAGTAGAGTCAACTTCATTCTT-3′. For puromycin, the respective sequences were forward 5′-ACCACGCCGGAGAGC-3′, reverse 5′-CCGGGAAC CGCTCAACTC-3′, and the reporter probe sequence was 5′-ACACCGCCCCCGCTTC-3′. Real-time qPCR analysis was carried out using the ViiA7 System (Applied Biosystems) and the amplification steps were as follows: 95°C for 10 min followed by 40 cycles of 95°C for 15 s and 60°C for 60 s. Relative quantification of genomic DNA was carried out with a comparative threshold cycle method using a TaqMan RNase P Control Reagents Kit (Thermo Fisher Scientific) for normalization.

### PCR, agarose gel electrophoresis, and sequence analysis

Fifty nanograms of genomic DNA were mixed with primers for the SDHB exon 2 knockout site (forward 5′-ATCCAGCGTTACATCTGTTGTGCCAGCAAAATGG-3′, reverse 5′-GTCTTGAACTCCCAACCTCAGATGATCTGCCCG-3′) and KOD Fx Neo (Toyobo, KFX-201) following the manufacturer's instructions. PCR was carried out with the following conditions: 2 min at 94°C, followed by 30 cycles of 15 s at 94°C, 30 s at 61°C, and then 30 s at 68°C. Amplified DNA fragments were separated by electrophoresis on a 2% agarose gel made with Agarose XP (Wako). The desired section of gel was cut out and purified with a QIAquick Gel Extraction Kit (Qiagen) following the manufacturer's instructions. Sequences of purified DNA were analyzed by Fasmac (Atsugi, Kanagawa, Japan).

### Proliferation testing of SDHB knockout cells

HCT116 control cells and HCT116 SDHB knockout #7 cells were plated at 3000 cells/100 μL/well on 96 well plates (Corning). From day 0 to day 4, cell viability was assessed with a Cell Titer-Glo Luminescent Cell Viability Assay (Promega) following the manufacturer's instructions.

### Cell growth assay and drug treatment

HCT116 control cells were plated at 2500 cells/100 μL/well and HCT116 SDHB knockout #7 and #23 cells were plated at 3500 cells/100 μL/well on 96 well plates (Corning) and cultured overnight. For the glucose deprivation experiments, the medium was changed to Roswell Park Memorial Institute (RPMI)-1640 supplemented with 10% FBS with the indicated concentration of glucose using RPMI-1640 (Wako, 189-02025) and glucose-free RPMI-1640 (Wako, 185-02865) 24 h post-seeding. After 4 days, cell viability was assessed with a Cell Titer-Glo Luminescent Cell Viability Assay (Promega). For drug sensitivity experiments, indicated concentrations of drugs were added 24 h post-seeding, and after 4 days, cell viability was assessed with a Cell Titer-Glo Luminescent Cell Viability Assay (Promega). Drugs used in this study were 2-deoxy-D-glucose (Sigma), phenformin (Wako), dehydroepiandrosterone (Tokyo Kasei), 5-pentadecylresorcinol (Sigma), CBR-5884 (Focus Biomolecules), NHI-2 (Focus Biomolecules), Compound 968 (synthesized as previously described [29]), CB-839 (Selleck Chemicals), JQ1 (synthesized as previously described [30]), I-BET151 (synthesized as previously described [31]), I-BET762 (synthesized as previously described [32]), and PFI-1 (synthesized as previously described [33]). Growth inhibition curves were plotted as percentages of control cells and growth inhibition of 50% values were determined by GraphPad Prism version 6 software (GraphPad Software, San Diego, CA, USA) by fitting a sigmoidal curve with a variable slope. For immunoblotting analysis, indicated concentrations of JQ-1 were added 24 h post-seeding, for 38 h.

### Western blotting

Cells were washed with phosphate buffered saline (PBS) at 4°C and lysed with cell lysis buffer comprising 62.5 mM Tris-HCl (Wako), 10% glycerol (Wako), and 1% sodium dodecyl sulfate (SDS) (Wako). After heating at 100°C for 5 min, the protein concentration was measured with a BCA Protein Assay Kit (Thermo Scientific). Lysates were prepared with 3-mercapto-1, 2-propanediol (Wako) and separated by SDS polyacrylamide gel electrophoresis (PAGE) using 7.5–15% SDS-PAGE gel (DRC). Proteins were electroblotted onto a polyvinylidene difluoride membrane (Wako) at 75 V for 120 min and blocked with Block Ace (DS Pharma Biomedical) in PBS containing 0.2% Tween 20 (PBS-T) or Starting Block T20 (PBS) Blocking Buffer (Thermo Scientific). Membranes were incubated with the specific primary antibody overnight at 4°C and washed three times with PBS-T. Membranes were incubated with horseradish peroxidase (HRP)-conjugated secondary antibody (eBioscience) for 1 h at room temperature, and then washed three times with PBS-T. The immunoblots were visualized by use of ImmunoStar Zeta (Wako) or ImmunoStar LD (Wako). Signals were visualized on an LAS-3000 image analyzer (Fujifilm) and quantified with Multi Gauge Ver. 3.1 (Fujifilm). The following antibodies were used: anti-SDHB (mouse monoclonal, abcam, ab14714, 1:5000), anti-SDHA (rabbit polyclonal, abcam, ab66484, 1:5000), anti-β-Actin (HRP conjugate, rabbit monoclonal, Cell Signaling Technology, #5125, 1:5000), anti-pyruvate dehydrogenase (rabbit polyclonal, Cell Signaling Technology, #2784, 1:5000), anti-pyruvate carboxylase (rabbit monoclonal, abcam, ab126707, 1:5000), anti-glutaminase 1 (mouse monoclonal, abcam, ab60709, 1:5000), anti-citrate synthase (rabbit monoclonal, Cell Signaling Technology, #14309, 1:5000), anti-isocitrate dehydrogenase 1 (IDH1) (rabbit monoclonal, Cell Signaling Technology, #8137, 1:5000), anti-oxoglutarate dehydrogenase (OGDH) (rabbit polyclonal, Cell Signaling Technology, #13407, 1:5000), anti-dihydrolipoamide S-succinyltransferase (DLST) (rabbit monoclonal, Cell Signaling Technology, #11954, 1:5000), anti-succinyl-CoA synthetase (rabbit polyclonal, Cell Signaling Technology, #5557, 1:5000), anti-fumarase (rabbit monoclonal, Cell Signaling Technology, #4567, 1:5000), anti-malate dehydrogenase 1 (MDH1) (rabbit monoclonal, abcam, ab181091, 1:5000), anti-MDH2 (rabbit monoclonal, Cell Signaling Technology, #11908, 1:5000), anti-glutamic-oxaloacetic transaminase 2 (GOT2) (rabbit polyclonal, protein tech, 14800-1-AP, 1:5000), anti-PHGDH (mouse monoclonal, abcam, ab57030, 1:1000), anti-PSAT1 (mouse polyclonal, Abnova Corporation, H00029968-A01, 1:1000), anti-phosphoserine phosphatase (PSPH) (rabbit polyclonal, Sigma, HPA020376, 1:1000), anti-c-Myc (rabbit monoclonal, Cell Signaling Technology, #5605, 1:5000), and anti-cleaved PARP (rabbit monoclonal, Cell Signaling Technology, #9541, 1:5000).

### Metabolomics analysis with capillary electrophoresis time-of-flight mass spectrometry

HCT116 control cells and HCT116 SDHB knockout #7 and #23 cells were plated at 8 × 10^5^ cells/10 mL in 10 cm dishes (Corning) and incubated for 48 h and 96 h. For metabolomics analysis after JQ-1 treatment, indicated concentrations of JQ-1 were added to the dishes 24 h post-seeding, and incubated for 38 h. Cells were washed with 5% D-mannitol (Wako). For metabolomics analysis of extracellular lactate, cells were incubated for 24 h post-seeding, and culture media were collected. Samples were extracted with methanol and purified with chloroform (Wako). Metabolites were analyzed with capillary electrophoresis time-of-flight mass spectrometry (CE-MS) in the laboratory of Dr. Soga as previously described [34].

### Stable isotope tracer experiments

HCT116 control cells or HCT116 SDHB knockout #7 and #23 cells were plated at 1 × 10^6^ cells/10 mL in 10 cm dishes (Corning) in RPMI-1640 with 10% FBS. The next day, the culture medium was replaced with glucose-free RPMI-1640 (Wako, 185-02865) supplemented with 10% FBS in the presence of 2 g/L [U-13C]-glucose (Cambridge Isotope Laboratories, Inc.) or glutamine-free RPMI-1640 (Wako, 183-02165) supplemented with 10% FBS in the presence of 300 mg/L [U-13C, 15N]-glutamine (Cambridge Isotope Laboratories, Inc.). The cells were incubated for the indicated time, washed with saline, and then extracted with methanol for the metabolic analysis using an Agilent 7200 Accurate-Mass Quadrupole time-of-flight gas chromatography–mass spectrometry system. The effects of natural isotopes were removed from the raw mass spectra for exact analysis of the labeling pattern. The ^13^C and ^15^N abundance of each labeled metabolite was calculated from the peak area as previously described [35].

### Measurement of oxygen consumption rate and extracellular acidification rate

HCT116 control cells or HCT116 SDHB knockout #7 and #23 cells were plated at 5000 cells/well on collagen-coated XF cell culture microplates (102601-100 Seahorse Bioscience) and incubated for 24 h. The medium was then replaced with XF Assay Medium (pH 7.4, Seahorse Bioscience, 102365-100) supplemented with 25 mM glucose, 1 mM sodium pyruvate, and 2 mM GlutaMAX. Cells were then placed in a CO_2_-free incubator for 1 h at 37°C. The ECAR and OCR were measured with an XF96 Extracellular Flux Analyzer (Seahorse Bioscience). The ECAR and OCR values were normalized in each well using the cell number measured with a CyQUANT Cell Proliferation Assay Kit (Thermo Fisher Scientific).

### RNA interference

For drug sensitivity experiments, Caki-2 cells were plated at 2000 cells/100 μL/well on 96 well plates and transfected with 10 nM of Silencer Select Predesigned siRNA targeting SDHB (siSDHB, s12653, Thermo Fisher Scientific) or Silencer Select Negative Control siRNA #2 (Thermo Fisher Scientific) using Dharmafect 1 Transfection Reagent (GE Healthcare Dharmacon) following the manufacture's instruction. Indicated concentrations of JQ-1 were added 48 h post transfection, and after 3 days, cell viability was assessed with a Cell Titer-Glo Luminescent Cell Viability Assay (Promega). For confirmation of knockdown efficiency of siSDHB, Caki-2 cells were plated at 6 × 10^4^ cells/2 mL/well on 6-well plates and transfected with 10 nM of siSDHB or negative control siRNA in the same manner. After 48 h, cells were harvested for western blotting.

### Statistical analysis

A *P*-value between HCT116 SDHB knockout cells #7 and control cells was calculated by the Student's *t*-test. Differences between the control and treatment groups in cell growth assays were analyzed by Williams’ test. A *P*-value of less than 0.05 for the Student's *t*-test and 0.025 for Williams' test was considered statistically significant. In consideration of multiple testing, *P*-values between HCT116 SDHB knockout cells #7, #23, and control cells were calculated by Bonferroni's corrected *t*-test.

## SUPPLEMENTARY MATERIALS FIGURES AND TABLES



## Abbreviations

2DG: 2-deoxy-glucose
ATP: adenosine triphosphate
BET: bromodomain and extra-terminal
CE-MS: capillary electrophoresis time-of-flight mass spectrometry
CRISPR: clustered regularly interspaced short palindromic repeat
DLST: dihydrolipoamide S-succinyltransferase
ECAR: extracellular acidification rate
FBS: fetal bovine serum
GOT2
HDR: homology directed repair
HIF: hypoxia inducible factor
HRP: horseradish peroxidase
IDH1: isocitrate dehydrogenase 1
MDH1: malate dehydrogenase 1
NADH: nicotinamide adenine dinucleotide
OCR: oxygen consumption rate
OGDH: oxoglutarate dehydrogenase
O-GlcNAc: O-linked β-N-acetylglucosamine
PAGE: polyacrylamide gel electrophoresis
PARP: poly-(adenosine diphosphate ribose) polymerase
PBS: phosphate buffered saline
PC: pyruvate carboxylase
PDH: pyruvate dehydrogenase
PHGDH: phosphoglycerate dehydrogenase
PSPH: phosphoserine phosphatase
PPP: pentose phosphate pathway
PSAT1: phosphoserine aminotransferase 1
qPCR: quantitative polymerase chain reaction
RPMI: Roswell Park Memorial Institute
SD: standard deviation
SDH: succinate dehydrogenase
SDS: sodium dodecyl sulfate
TCA: tricarboxylic acid
UDP: uridine diphosphate
