# Supplementary material for: Succinate dehydrogenase B-deficient cancer cells are highly sensitive to bromodomain and extra-terminal inhibitors
Source: Oncotarget. 2017 Mar 7;8(17):28922–38. doi: 10.18632/oncotarget.15959 (PMC5438703; doi:10.18632/oncotarget.15959)
Supplement: Supplementary file 1 [file oncotarget-08-28922-s001.pdf]

Succinate dehydrogenase B-deficient cancer cells are highly sensitive to bromodomain and extra-terminal inhibitors

SUPPLEMENTARY FIGURES

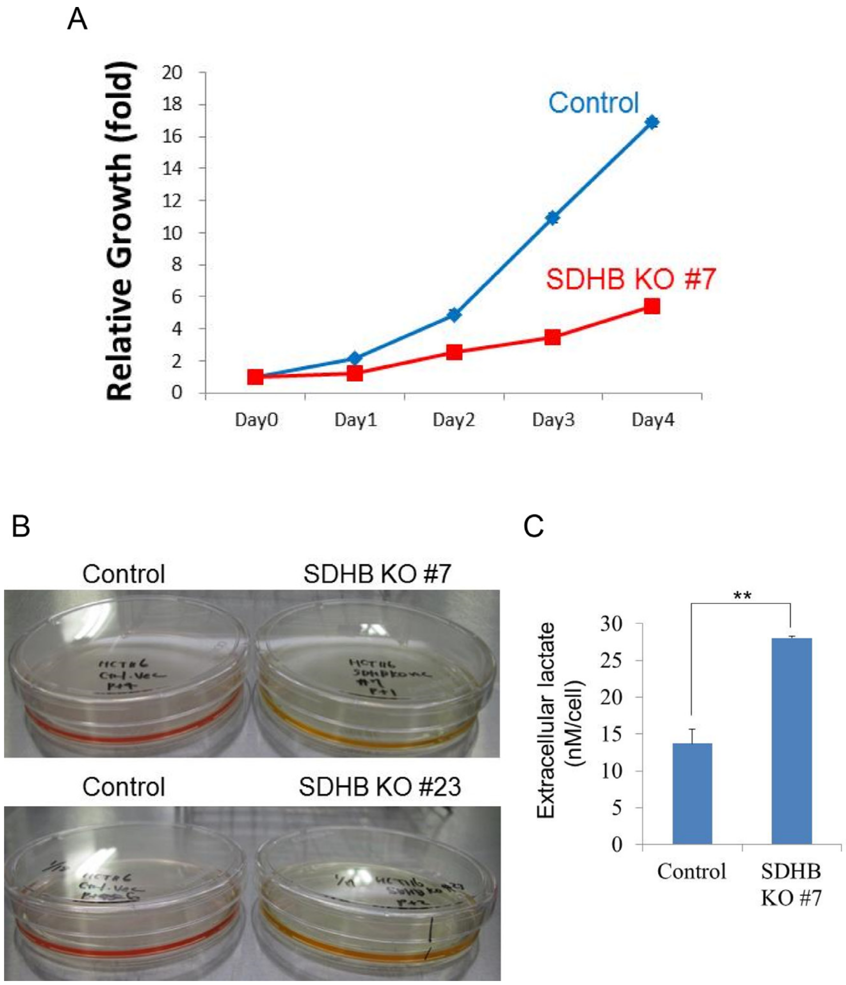

Supplementary Figure 1: Slower growth of SDHB knockout cells dependent on glycolysis. (A) SDHB knockout #7 cells and control cells were seeded and cultured for the indicated number of days. Relative cell growth was assessed by setting the cell number at Day 0 as 1. Data are given as means ± SD (n = 3). **(B)** Photo of SDHB knockout cells and control cells cultured to 100% confluence. **(C)** Lactate concentrations in the medium were measured 24 h after seeding of SDHB knockout cells (#7) and control cells. Data are presented as means ± SD; *P < 0.05, **P < 0.01, ***P < 0.001 by the Student's *t*-test.

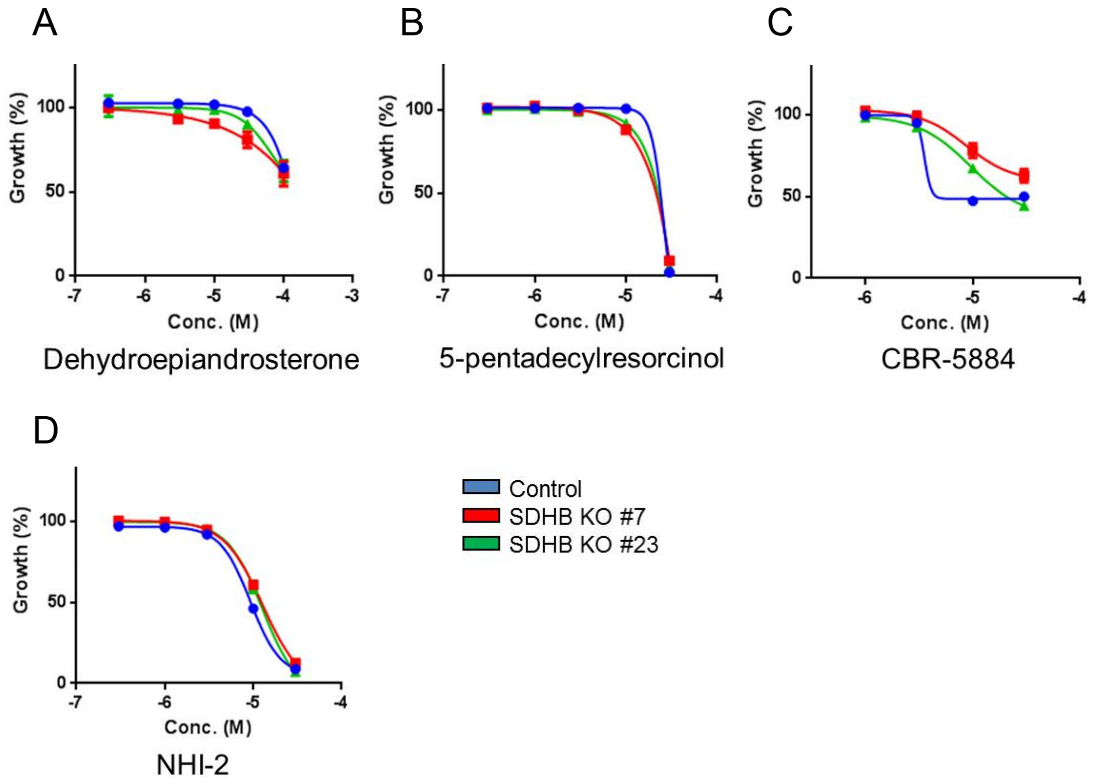

Supplementary Figure 2: Sensitivities of SDHB knockout cells to inhibitors of glycolysis and its derived pathway. (A-D) SDHB knockout cells and control cells were treated with the indicated concentrations of the glucose-6-phosphate dehydrogenase inhibitor dehydroepiandrosterone, the glycerol-3-phosphate dehydrogenase 1/2 inhibitor 5-pentadecylresorcinol, the PHGDH inhibitor CBR-5884, and the lactate dehydrogenase A inhibitor NHI-2. After 4 days, cell viability was assessed. Ordinate values were obtained by setting the vehicle control value as 100%. Data are given as means ± SD (n = 3).

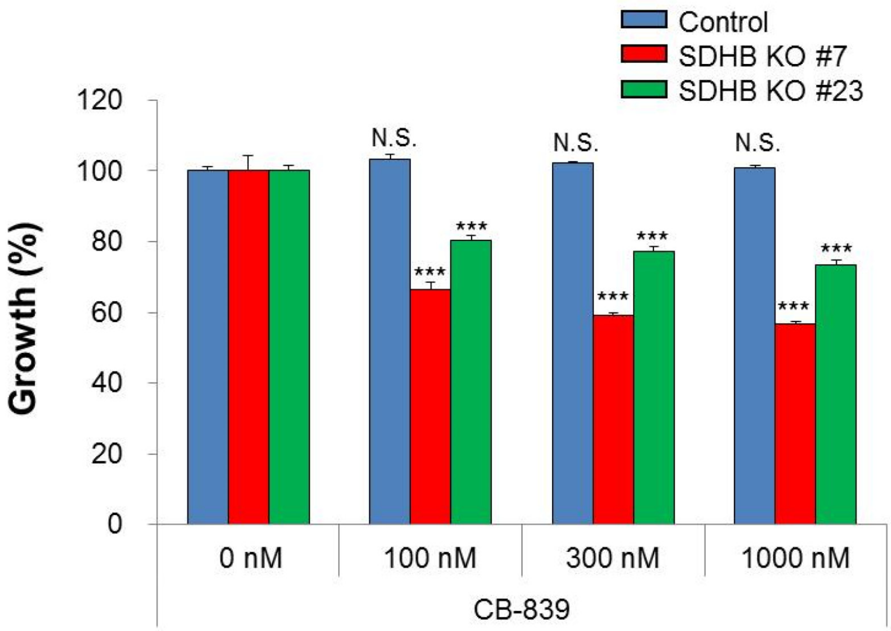

Supplementary Figure 3: Susceptibility of SDHB knockout cells to selective glutaminase 1 inhibitor CB-839. HCT116 control cells and HCT116 SDHB knockout #7 and #23 cells were treated with the indicated concentration of CB-839. After 4 days, cell viability was assessed. Ordinate values were obtained by setting the control group value as 100%. Data are presented as means ± SD (n = 3). N.S., not significant. *P < 0.025, **P < 0.005, ***P < 0.0005 by Williams' test.

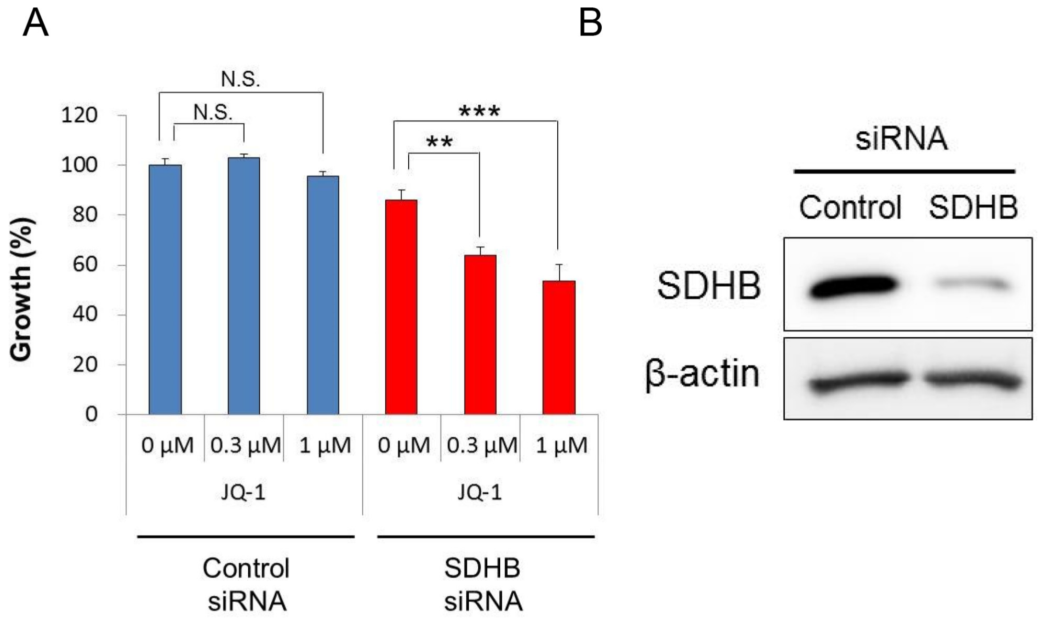

Supplementary Figure 4: SDHB knockdown enhanced the susceptibility of the renal cell carcinoma cell line Caki-2 to the BET inhibitor JQ-1. (A) Caki-2 cells were treated with siRNA targeting SDHB or negative control siRNA (control siRNA). After 48 h, cells were treated with the indicated concentrations of JQ-1. After 3 days, cell viability was assessed. Ordinate values were obtained by setting the control group value as 100%. Data are presented as means ± SD (n = 3); N.S., not significant, *P < 0.025, **P < 0.005, ***P < 0.0005 by Williams' test. **(B)** Caki-2 cells transfected with siRNA targeting SDHB or control siRNA were lysed after 48 h, and expression levels of SDHB and β-actin were determined by western blotting.

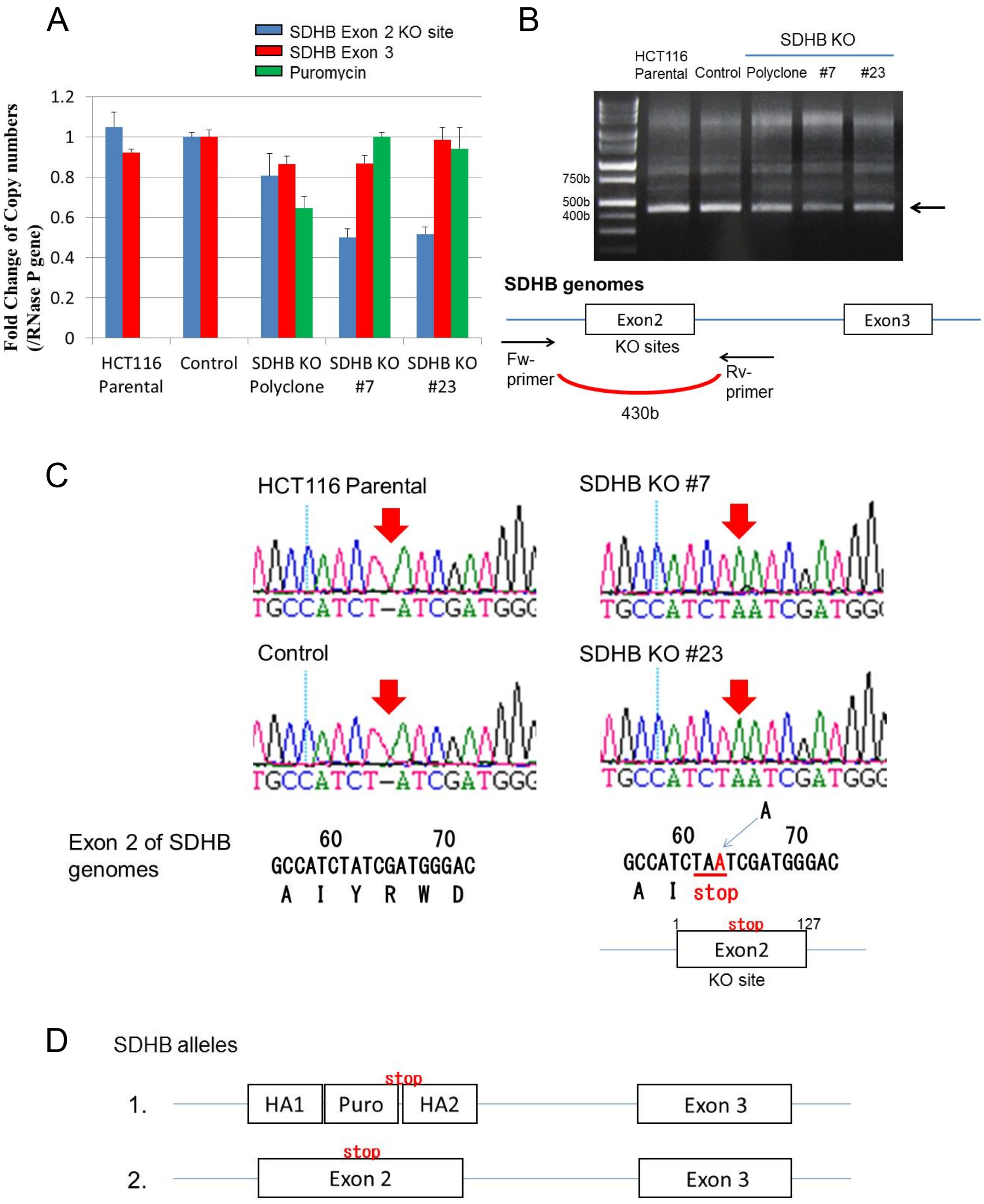

Supplementary Figure 5: Establishment of SDHB knockout cells. **(A)** Copy numbers of the SDHB genomic locus and the puromycin resistance gene in HCT116 parental cells, control cells (HCT116 cells transfected with a control vector), SDHB knockout polyclonal cells, and SDHB knockout #7 and #23 cells were examined by qPCR. Fold change of copy number was assessed by setting the control vector-transfected control group value as 1. Data are given as means ± SD. **(B)** PCR analysis of the SDHB exon 2 knockout site of genomic DNA was performed. The schematic illustration shows the sites of the SDHB genome amplified by PCR. **(C)** Sequenced data in exon 2 of the SDHB genome are shown. An adenine insertion between the 62nd and the 63rd base in exon 2 of SDHB formed a termination codon specifically in SDHB knockout #7 and #23 cells. **(D)** The diagram shows SDHB alleles in SDHB knockout #7 and #23 cells.
